# Supplementary material for: Bright semiconductor single-photon sources pumped by heterogeneously integrated micropillar lasers with electrical injections
Source: Light Sci Appl. 2023 Mar 6;12:65. doi: 10.1038/s41377-023-01110-9 (PMC9986240; doi:10.1038/s41377-023-01110-9)
Supplement: Supplementary file 1 — Supplementary Information Bright Semiconductor Single-Photon Sources Pumped by Heterogeneously Integrated Micropillar lasers with Electrical injections [file 41377_2023_1110_MOESM1_ESM.docx]

**Supplementary Information for**

**Bright Semiconductor Single-Photon Sources Pumped by Heterogeneously Integrated Micropillar lasers with Electrical Injections**

Xueshi Li^1,*^, Shunfa Liu^1,*^, Yuming Wei^1^, Jiantao Ma^1^, Changkun Song^1^, Ying Yu^1^, Rongbin Su^1^, Wei Geng^2^, Haiqiao Ni^3,4^, Hanqing Liu^3,4^, Xiangbin Su^3,4^, Zhichuan Niu^3,4,†^, Youling Chen^3,4,‡^, and Jin Liu^1,§^

^1^ State Key Laboratory of Optoelectronic Materials and Technologies, School of Physics, School of Electronics and Information Technology, Sun Yat-sen University, Guangzhou 510275, China

^2^ Hisilicon Research, Huawei Techologies Co., Ltd, Shenzhen 518129, China

^3^ State Key Laboratory for Superlattice and Microstructures, State Key Laboratory on Integrated Optoelectronics, Institute of Semiconductors, Chinese Academy of Sciences, Beijing 100083, China

^4^ Center of Materials Science and Optoelectronics Engineering, University of Chinese Academy of Sciences, Beijing 100049, China

* These authors contributed equally

† [zcniu@semi.ac.cn](mailto:zcniu@semi.ac.cn)

‡ [ylchen@semi.ac.cn](mailto:ylchen@semi.ac.cn)

§ [liujin23@mail.sysu.edu.cn](mailto:liujin23@mail.sysu.edu.cn)

**Ⅰ. CHARACTERIZATION OF THE QD WAFER**

The QD wafer is schematically shown in Fig.S1(a), consisting of a 160 nm-thin GaAs membrane containing InAs quantum dots (QDs), a sacrificial layer (1 μm Al_0.8_Ga_0.2_As) and a GaAs substrate. Fig.S1(b) shows the high-resolution high-angle annular dark field (HAADF) STEM images along [110] crystallographic direction of single InAs QD. The bright regions close to the center of the image represent the InAs QDs, with the average height of 6 nm. The AlGaAs capping layer can be clearly presented as the darker region surrounding the QD. A representative spectrum of single QD is shown in Fig.S1(c).

Fig.S1: Characterization of the QD wafer. (a) Schematics of the QD wafer. (b) STM image of the InAs QDs used in the work. (c). A representative PL spectrum for the single QD.

**Ⅱ. FABRICATION FLOW OF THE CBG**

The full fabrication flow of deterministically coupled QD-CBG is shown in Fig.S2(a). The process starts with the III-V wafer consists of a 160 nm-thin GaAs membrane containing InAs quantum dots (QDs) grown on top of a sacrificial layer (1 μm Al_0.8_Ga_0.2_As) and a GaAs substrate. To acquire the positions of QDs, firstly, metallic alignment marks are created on the surface of the sample with standard E-beam lithography, metal deposition and lift-off processes. Then, the positions of the QDs respective to the alignment marks are extracted from the wide-field PL images. After that, the shallow etched CBGs with QD in the center are fabricated through another aligned E-beam lithography followed by a chlorine-based dry etch process. After removing the photoresist by oxygen plasma surface treatments, we dip the sample in the acid solution of HF 10% for the necessary time to remove the sacrificial layer. We finally obtain the suspended deterministically coupled QD-CBG devices by drying the sample in isopropanol, as shown in Fig.S2(b). The parameters of CBG during fabrication are carefully chosen to ensure the spectral matching between the X state of the QD and the fundamental cavity mode of the CBG.

Fig.S2: Fabrication flow of deterministically coupled QD-CBG devices.

**Ⅲ. FABRICATION FLOW OF THE MICROPILLAR LASER**

The full fabrication flow of the micropillar laser is shown in Fig.3(a). The wafer consists of a single layer GaAs with QW between 23(30) top(bottom) GaAs/Al_0.9_Ga_0.1_As distributed Bragg reflector (DBRs) provided by EPIHOUSE. In order to achieve an electrically pumped micropillar laser, we need to properly dop the wafers. The epitaxial n-type and p-type regions are realized by doping the GaAs during the growth with silicon and carbon, respectively. The first step in creating the micropillar laser is the fabrication of electrical contacts to the n-doped layers. We obtain the Ni/Ge/Au/Ni/Au contacts on the back of the wafer by E-beam evaporation, followed by an annealing process. Then, the masks of the electrically-injected micropillar lasers and alignment marks are fabricated in a specific array based on the relative positions of fabricated CBGs by using an E-beam lithography. After the chlorine-based dry etch process, we use BCB to flatten the micropillars to ensure that the upper surfaces of the micropillars are just exposed. This process requires multiple spin-coatings of the photoresist, annealing and dry etching processes. Finally, the positive electrode is created by using an E-beam lithography, metal evaporation and lift-off processes. The SEM images of the samples are shown in Fig.S3(b).

Fig.S3: Fabrication flow of micropillar laser.

**Ⅳ. TRANSFER PRINTING PROCESS FOR MULTIPLE DEVICES**

After the fabrications of the CBGs, we realize the micropillars at the corresponding positions according to the layout of the CBG array. Therefore, during the transfer process, we can align multiple CBGs with micropillars to achieve potentially scalable device integrations. We show three of 8 transferred devices at one time in Fig.S4(a,b). The extraction efficiencies of each device are listed in Fig.S4(c), featuring high yields for the multiple device transfer printing process.

Fig.S4: Transfer printing process for multiple devices.

We further present the transferring of a large array of devices in Fig.S5. 20 out of 26 devices are successfully fabricated in a single run. The fabrication throughput and yield could be further boosted by exploiting commercially available tools that are specially designed for the transfer printing process.

Fig.S5: The result of a large scale transfer printing process.

**Ⅴ. NUMERICAL SIMULATIONS OF CBG**

The design of CBG for QD single-photon sources has been thoroughly discussed in the literature^1,2^. Here we briefly show the optimization of the parameters in Fig.S(6). We use the central disk with a radius of 2p in which p and w are the periodicity and the width of the etched trenches respectively, as shown in Fig.S6(a). The cavity modes in XY plane and YZ plane are plotted in Fig.S6(b,c) respectively. In Fig.S6(d), the cavity resonances for different p are shown. To match the QD emission wavelength (~907 nm), we chose p=335 nm. We further change w in Fig.S6(e), and w=110 nm gives rise to the highest Purcell factor at the targeted wavelength. Finally, the extraction efficiency for the optimized parameter (p=335 nm and w=110 nm) as a function of NA is presented in Fig.S6(f).

Fig.S6: Simulations of the CBG. (a) Schematics of the CBG. (b) Cavity mode profile in XY plane. (c) Cavity profile in YZ plane. (d) Spectra of cavity modes with different p. (e) Spectra of cavity modes with different w. (f) Extraction efficiency as a function of the NA for the CBG with p=335 nm and W= 110 nm. The star denotes the NA used in our experiment.

We further present the Purcell factor and extraction efficiency (with NA=0.65) as a function of the placement deviation and fabrication resolution in Fig.S7 and Fig.S8, respectively. The displacement refers the distance from QD to the center of the CBG. The fabrication resolution mostly reflects on the width of the etched trenches. Due to the uncertainty of the dipole orientation, we have simulated both x-polarized and y-polarized dipoles. The Purcell factors and extraction efficiencies of other orientated dipoles fall into the range between the x-polarized and y-polarized dipoles^2^. Compared to the simulations, our QD seems to be within 300 nm from the center of the CBG, which is probably due to an unintentional offset in the aligned electron beam lithography process.

Fig.S7: Purcell factor as a function of etched trench width and placement deviation inside the CBG. (a) Results for an x-oriented dipole; (b) Result for a y-oriented dipole; (c) Maximum achievable Purcell factor; (d) Minimum achievable Purcell factor; (e) Purcell factor as a function of dipole displacement from the CBG with w=100 nm and w=110 nm [shown as dashed lines in (a)-(b)]. Shaded areas correspond to the uncertainty in Purcell factor due to lack of knowledge of the dipole orientation. The dashed line corresponds to an average value.

Fig.S8: Extraction efficiency (NA=0.65) as a function of etched trench width and placement deviation inside the CBG. (a) Results for an x-oriented dipole; (b) Results for a y-oriented dipole; (c) Maximum achievable extraction efficiency; (d) Minimum achievable extraction efficiency; (e) Extraction efficiency as a function of dipole displacement from the CBG with w=100 nm and w=110 nm [shown as dashed lines in (a)-(b)]. Shaded areas correspond to the uncertainty in extraction efficiency due to lack of knowledge of the dipole orientation. The dashed line corresponds to an average value.

**Ⅵ. CHARACTERIZATIONS OF MICROLASERS**

We’ve presented the far-field pattern of the microlaser in Fig.S9. The far-field emission is close to an Gaussian profile with a divergence angle below 10 degree.

Fig.S9: Beam profile of the microlaser. (a) Far-field pattern of the electrically injected micropillar laser. (b) y-cut of (a), exhibiting a divergence angle below 10 degree.

**Ⅶ. ESTIMATION OF EXTRACTION EFFICIENCY**

The extraction efficiency of the hybrid SPS at the first lens is evaluated by externally exciting the device using a femto-second pulsed laser with a repetition rate of 79.6 MHz. Fig.S10(a) shows the schematic of the experimental setup for estimating the extraction efficiency. The emitted photons are collected using a microscope objective with an NA of 0.65. For this measurement, the signals pass through a dichroscope (DM), a beam splitter (BS), a long pass filter (LPF), and a bandpass filter(BPF) before coupling into a single-mode fiber. The detection efficiency of the whole setup has been carefully calibrated to be 0.0405, as shown in Fig.S10(b). A saturation count rate of 0.82 MHz was extracted from Fig.S10(c), which results in an extraction efficiency at the first lens of 25.44%.

Fig.S10: Estimation of the extraction efficiency at the first lens. (a) Setup for the extraction efficiency measurement. (b) Transmissions of the elements in the setup. (c) Photon count rates as a function of the pulsed laser power.

**Ⅷ. SCHEME FOR ON-CHIP RESONANT EXCITATION**

Inspired by recent works of exploiting asymmetrical microcavities for resonance fluorescence^3,4^, we propose to employ an elliptical micropillar for obtaining a single-mode laser with H-polarization, as shown in Fig.S11(a). Meanwhile an asymmetrical CBG is employed in which a charged exciton is resonant with V-polarized cavity mode and the tail of H-polarized cavity mode is used for the resonant excitation, as shown in Fig.S11(b). In such a configuration, an external polarizer along V direction as shown in Fig.S11(c) will remove the H-polarized laser background and result in V-polarized single photons with a high indistinguishability.

Fig.S11: Implementation of on-chip resonant excitations in our platform. (a) H-polarized lasing mode from an elliptical micropillar. (b) Spectral alignment for QD and cavity modes of an asymmetrical CBG for the resonant excitation. (c) Schematic of the on-chip resonant excitation scheme.

**Ⅸ. COMPARISON TO DEVICES IN THE LITERATURE**

We’ve compared our devices with representative of the state-of-the-art in Table 1. Compared to the state-of-the-art devices under on-chip excitation, our device shows a much better single-photon purity and a higher extraction efficiency. However, the best reported devices under off-chip excitation still exhibit better metrics in terms of extraction efficiency, single-photon purity as well as the indistinguishability. We believe that the performance of our device could be further significantly improved with optimizations of the fabrication process and implementations of on-chip resonant excitation in Fig.S11.

Fig.S12: Comparison to the devices in the literature.

**Reference:**

1 Davanço, M. *et al*. A circular dielectric grating for vertical extraction of single quantum dot emission. *Applied Physics Letters* **99**, 041102 (2011).

2 Sapienza, L. *et al*. Nanoscale optical positioning of single quantum dots for bright and pure single-photon emission. *Nature Communications* **6**, 7833 (2015).

3 Wang, H. *et al*. Towards optimal single-photon sources from polarized microcavities. *Nature Photonics* **13**, 770-775 (2019).

4 Gerhardt, S. *et al*. Polarization-dependent light-matter coupling and highly indistinguishable resonant fluorescence photons from quantum dot-micropillar cavities with elliptical cross section. *Physical Review B* **100**, 115305 (2019)
